# Identification of novel peripherally restricted risperidone-like dopamine D2 receptor antagonists for prolactin-mediated neuroprotection through a multi-stage *in silico* screening

**DOI:** 10.1371/journal.pone.0355997

**Published:** 2026-08-28

**Authors:** Shinhyun Kim, Won Hee Jo, Jae Ok Cha, Min-Sun Kim

**Affiliations:** College of Pharmacy and Research Institute of Life and Pharmaceutical Sciences, Sunchon National University, Sunchon, Jeonnam, Republic of Korea; Bai Jerbai Wadia Hospital for Children, INDIA

## Abstract

Prolactin, a hormone with emerging neuroprotective roles, has demonstrated potential in mitigating cognitive impairment under specific neurotoxic conditions, including environmental toxicity and excitotoxicity. While dopamine D2 receptor (D2R) antagonists, such as risperidone, can elevate systemic prolactin levels to exert beneficial effects in the central nervous system (CNS), their clinical repurposing is constrained by CNS-mediated side effects associated with blood–brain barrier (BBB) penetration. In this study, we aimed to identify novel D2R antagonist candidates predicted to have restricted BBB penetration and the potential to stimulate prolactin secretion without substantial CNS exposure, thereby reducing central side effects including extrapyramidal symptoms and sedation. A systematic multi-stage in silico screening workflow was implemented. Ligand-based virtual screening using SwissSimilarity against the ZINC-20 database identified 400 structural analogs of risperidone. SwissADME-based ADMET filtering selected 226 candidates predicted to be BBB-impermeable, from which molecular docking using AutoDock Vina identified eight compounds with docking scores more favorable than that of risperidone. Subsequent interaction analysis identified four candidates that maintained critical contacts with Asp80 (3.32) and Trp373 (6.48), key residues involved in D2R ligand binding. Molecular dynamics simulations over 100 ns were used to assess conformational behavior, with Compound #128 exhibiting the most favorable ligand stability profile among the tested candidates and showing behavior broadly comparable to that of risperidone. These findings suggest that the identified compounds may serve as computationally prioritized candidates for the development of peripherally restricted D2R antagonists. However, their functional D2R antagonism, actual BBB exclusion, prolactin-elevating activity, and ability to reproduce CNS neuroprotection without direct central D2R modulation remain to be experimentally validated.

## Introduction

Prolactin is an evolutionarily conserved polypeptide hormone classically known for its essential roles in lactation and breast development [1]. However, emerging evidence indicates that it modulates a broad spectrum of physiological processes, including reproduction, immunomodulation, angiogenesis, osmotic balance, and glucose metabolism [2,3]. Notably, within the central nervous system (CNS), prolactin has been increasingly recognized as a potent neuroprotective factor. It exerts important effects on neuronal survival, synaptic plasticity, and neurogenesis in distinct brain regions. For instance, depending on sex and specific physiological conditions, it promotes neurogenesis in the forebrain subventricular zone of pregnant animals [4,5] and in the hippocampal dentate gyrus of chronically stressed adult male mice [6]. Beyond these physiological roles in CNS, prolactin exerts potent neuroprotective effects against severe neurological insults, particularly glutamate and kainic acid-induced excitotoxicity, via the PI3K/AKT, NF-κB, and JAK2/STAT5 signaling pathways [7–9]. In primary hippocampal neuronal cultures, prolactin directly activates its cognate receptors to attenuate excitotoxicity through multiple mechanisms, including calcium homeostasis regulation, anti-apoptotic signaling, and antioxidant defense [10–12]. Furthermore, prolactin upregulates key gene networks associated with learning, memory, neurodevelopment, and plasticity within the hippocampus [13].

Building upon this neuroprotective foundation, our previous studies demonstrated that strategically elevating systemic prolactin levels can prevent cognitive impairments under specific neurotoxic conditions. We found that the administration of dopamine D2 receptor (D2R) antagonists such as risperidone and amisulpride markedly stimulated prolactin secretion, which in turn effectively ameliorated neurotoxicity and cognitive deficits induced by 1,2-diacetylbenzene (DAB) in vitro and in vivo [14,15]. Mechanistically, this prolactin-mediated neuroprotection was driven by the activation of multiple pro-survival signaling cascades, including the JAK/STAT, PI3K/AKT, and role of prolactin in the BDNF/ERK/CREB pathways, which mitigated DAB-induced pathological events, including tau hyperphosphorylation and oxidative stress. This highlights the therapeutic potential of targeting the prolactin axis for cognitive preservation against neurotoxicity caused by organic solvents, especially DAB.

Despite these benefits in the CNS, the clinical repurposing of antipsychotics with D2 antagonism for neuroprotection is severely constrained. Because these antipsychotics readily cross the blood-brain barrier (BBB), they frequently induce various CNS side effects including weight gain, sedation, and extrapyramidal symptoms by blocking central D2Rs [16–19]. To retain the neuroprotective benefits of antipsychotics while avoiding CNS side effects, there is potential to strategically take advantage of the anatomical feature that prolactin-secreting pituitary lactotrophs are situated outside the BBB. This distinct positioning implies that dopaminergic signaling in lactotrophs can be selectively modulated by peripherally restricted D2 antagonists to enhance prolactin levels without interfering with central dopaminergic neurotransmission or triggering extrapyramidal symptoms.

Among the second-generation antipsychotics, risperidone was selected as the prototype scaffold for this study based on the following rationale: (i) our previous work has demonstrated that risperidone-induced prolactin elevation confers measurable neuroprotective effects in animal models of neurotoxicity [14]; (ii) risperidone exhibits one of the highest prolactin-elevating potencies among D2 antagonists [20]; and (iii) its QT prolongation risk is classified as management of intermediate [21], which is a manageable concern in the design of peripherally restricted derivatives.

Importantly, the availability of high-resolution structural data has provided a robust foundation for structure-based drug design targeting the D2 receptor. The crystal structure of the human D2R in complex with risperidone (PDB: 6 CM4) has been resolved in detail, enabling precise identification of key interacting residues and binding modes through site-directed mutagenesis studies [22]. Leveraging this structural information, pharmacophore modeling coupled with molecular docking and molecular dynamics (MD) simulations has been widely employed in drug repositioning and target-based screening studies. For example, Mejía-Gutiérrez et al. (2021) utilized the 6 CM4 structure to screen a broad range of dopaminergic compounds beyond risperidone, identifying repurposable candidates for schizophrenia treatment [23]. Similarly, Mansoor and Morra et al. (2025) performed comparative MD analyses of structurally diverse D2R ligands including partial agonists such as aripiprazole and sulpiride, revealing distinct conformational dynamics upon ligand binding [24]. Nevertheless, risperidone is a multi-target aminergic receptor antagonist that interacts not only with D2R and 5-HT2A but also with H1 and α1 adrenergic receptors [25]. Because H1 and α1 adrenergic receptor interactions are associated with off-target liabilities such as sedation, metabolic effects, and orthostatic hypotension, receptor selectivity should be considered when designing risperidone-derived peripheral D2R antagonists [26]. Accordingly, 5-HT2A, H1, and α1 adrenergic receptors were included as representative receptors for preliminary selectivity docking, with D2R binding retained as the primary selection criterion [27–29].

Building on this theoretical and structural foundation, we implemented a systematic multi-stage virtual screening workflow integrating ligand-based similarity screening, pharmacokinetic profiling for BBB impermeability, molecular docking, and molecular dynamics simulations to identify optimal candidates for a safe and effective peripheral prolactin-elevating agent. Through this approach, we sought to obtain novel peripherally restricted analogs derived from the risperidone scaffold that would stimulate prolactin secretion to achieve potent neuroprotective effects while circumventing CNS-mediated side effects inherent to conventional BBB-permeable antipsychotics.

## Materials and methods

### Small molecules selection

To identify novel structural analogs with potential pharmacological activity, we utilized the Expasy SwissSimilarity platform [30]. The chemical structure of risperidone was employed as the query molecule to screen the ZINC-20 database [31]. SwissSimilarity performs ligand-based virtual screening using multiple approaches, including 2D molecular fingerprints and 3D electroshape vectors, to rapidly identify small molecules with high structural similarity to the query compounds. From the screening results, top-ranked hits were retrieved based on their similarity scores for further analysis.

### In silico ADME profiling and BBB permeability analysis

The pharmacokinetic profiles and drug-likeness of the selected analogs were evaluated using the SwissADME web tool [32]. Initial filtering was conducted based on Lipinski’s Rule of Five (RO5) to ensure favorable oral bioavailability properties, including molecular mass (< 500 Da), hydrogen bond donors (≦ 5), hydrogen bond acceptors (≦ 10), and calculated lipophilicity (MlogP ≦ 4.15) [33]. A key criterion in this study was to identify peripherally acting antagonists that minimize central nervous system (CNS) side effects, similar to the pharmacokinetic profile of domperidone, unlike the parent antipsychotics (haloperidol, amisulpride, olanzapine, and risperidone) which readily cross the blood-brain barrier (BBB). To achieve this, we utilized the BOILED-Egg model implemented in SwissADME, pkCSM, ADMETlab 3.0 [32,34–36]. SwissADME predictive model employs two key physicochemical descriptors, WLOGP (for lipophilicity) and TPSA (for apparent polarity), to estimate passive gastrointestinal absorption and brain access. pkCSM was used to obtain BBB permeability expressed as logBB, CNS permeability expressed as logPS, and categorical P-glycoprotein substrate prediction. ADMETlab 3.0 was used to obtain the predicted probability of BBB penetration and P-glycoprotein substrate classification. Compounds predicted to have high gastrointestinal absorption (HIA) but low BBB permeability (outside the yolk of the BOILED-Egg) were prioritized. This filtering step allowed for the exclusion of molecules likely to penetrate the CNS, and the resulting BBB-impermeable candidates were selected for subsequent molecular docking simulations.

### Molecular docking

The crystal structure of the human dopamine D2R in complex with risperidone was retrieved from the Protein Data Bank (PDB ID: 6 CM4) [22]. To repair missing atoms and discontinuous loop regions in the crystallographic structure, a complete 3D receptor model was generated using the Swiss-Model server [37]. To evaluate the binding affinity of the selected BBB-impermeable candidates toward the prepared dopamine D2 receptor (D2R) model, molecular docking simulations were performed using co-crystalized risperidone (8NU) as the reference standard. All protein and ligand structures were prepared and converted into. pdbqt format prior to analysis. The docking computations were carried out using AutoDock Vina v1.2.7 [38], utilizing the iterated local search global optimizer algorithm with a flexible side chain protocol. The docking site was defined based on the location of the co-crystallized ligand (8NU) within the D2R structure. The grid box was centered at coordinates x = 10 Å, y = 5.9 Å, and z = −9.5 Å, with dimensions of 30 × 30 × 30 Å to provide sufficient space for the ligands to rotate freely. The docking parameters were set with an exhaustiveness of 16, a maximum number of 9 binding modes, and an energy range of 3 kcal/mol. The co-crystallized risperidone ligand (8NU) in 6 CM4 was used for docking validation and affinity comparison.

The crystal structures of the human 5-HT2A receptor in complex with risperidone (PDB ID: 6A93) [27], human histamine H1 receptor in complex with doxepin (PDB ID: 3RZE) [28], and human α1B adrenergic receptor in complex with the inverse agonist (+)-cyclazosin (PDB ID: 7B6W) [29] were retrieved from the PDB. For each receptor, the docking site was defined based on the location of the corresponding co-crystallized ligand within the receptor structure, as this position represents the experimentally resolved orthosteric ligand-binding pocket. The grid centers were set as follows: 5-HT2A receptor, x = 15.024 Å, y = 0.273 Å, and z = 39.004 Å, based on the co-crystallized risperidone ligand (ligand ID: 8NU); H1 receptor, x = 16.875 Å, y = 36.082 Å, and z = 22.004 Å, based on the co-crystallized doxepin ligand (ligand ID: 5EH); and α1B adrenergic receptor, x = 1.589 Å, y = 27.078 Å, and z = −11.468 Å, based on the co-crystallized (+)-cyclazosin ligand (ligand ID: T0B).

Post-docking analysis and visualization of protein-ligand interactions in 2D and 3D were performed using BIOVIA Discovery Studio Visualizer. Of note, the homology model generated by Swiss-Model involved truncation of the N-terminal region relative to the original crystal structure (PDB: 6 CM4), resulting in a shift in residue numbering. Sequence alignment confirmed that Asp114 and Trp386 in the full-length D2R sequence correspond to Asp80 and Trp373 in our model, respectively. These residues represent the conserved Ballesteros-Weinstein positions 3.32 and 6.48 and are denoted as Asp80 (3.32) and Trp373 (6.48) throughout the manuscript. For clarity, the correspondence among full-length D2R numbering, model-based numbering, and Ballesteros-Weinstein notation is summarized in Table 1.

**Table 1:** Residue numbering correspondence for key D2R binding-site residues.

Functional role	Full-length D2R numbering	Model-based numbering used in this study	Ballesteros–Weinstein notation
Conserved Asp in orthosteric site	Asp114	Asp80	3.32
Hydrophobic pocket residue	Cys118	Cys84	3.36
Hydrophobic pocket residue	Thr119	Thr85	3.37
Hydrophobic pocket residue	Ser197	Ser163	5.46
Hydrophobic pocket residue	Phe198	Phe164	5.47
Hydrophobic pocket residue	Phe390	Phe376	6.44
Conserved Trp / toggle-switch residue	Trp386	Trp373	6.48
Hydrophobic pocket residue	Phe391	Phe377	6.52

Residue numbering differs between the full-length human D2R sequence and the Swiss-Model-generated receptor model because the N-terminal region was truncated during model preparation. The model-based numbering is used throughout this manuscript, with Ballesteros–Weinstein notation provided in parentheses.

### Key interaction analysis

Post-docking analysis and visualization of protein-ligand interactions in 2D and 3D were performed using BIOVIA Discovery Studio Visualizer. Importantly, the recent elucidation of the high-resolution crystal structure of the human D2R in complex with risperidone (PDB: 6 CM4) has provided a robust foundation for structure-based drug design, serving as the structural blueprint for our filtering strategy [22]. According to this crystal structure, the binding mode is fundamentally driven by two main structural features. First, a critical salt-bridge interaction is formed between the tertiary amine of risperidone and Asp80 (3.32). Second, the benzisoxazole moiety of risperidone extends into a deep hydrophobic pocket located below the orthosteric site. This distinct subpocket is defined by a network of seven key residues comprising Cys84 (3.36), Thr85 (3.37), Ser163 (5.46), Phe164 (5.47), Phe376 (6.44), Trp373 (6.48), and Phe377 (6.52). To select optimal candidates for subsequent MD simulations, the docking hits were rigorously filtered based on their ability to maintain these critical interactions with the established D2R orthosteric binding site. Specifically, visual inspection was conducted to confirm the formation of the salt-bridge with Asp(3.32) and engagement with the deep hydrophobic pocket, particularly focusing on the key residue Trp(6.48). Since previous mutational studies established that the loss of interaction with these pivotal residues abolishes binding activity (Ki > 10,000 nM), this structural criterion was strictly applied [22,39]**.** Within our generated 3D receptor model, these essential Ballesteros-Weinstein positions, Asp(3.32) and Trp(6.48), correspond to Asp80 and Trp373, respectively. Candidates that exhibited superior binding affinity compared to the reference drug while successfully preserving these critical contacts were advanced to the next validation stage.

### Molecular dynamics simulations

Molecular dynamics (MD) simulations were performed to investigate the stability and conformational dynamics of the prioritized ligand-protein complexes in a realistic physiological environment. The simulation systems were prepared using the CHARMM-GUI Membrane Builder [40]. The protein-ligand complexes were embedded in a bilayer of approximately 256 POPC (1-palmitoyl-2-oleoyl-sn-glycero-3-phosphocholine) and solvated with TIP3P water molecules [41,42]. Neutralizing ions were added, and the salt concentration was adjusted to 0.15 M NaCl to mimic physiological conditions. The CHARMM36m force field was applied for the protein and lipids, while the ligand topology and parameters were generated using the CHARMM General Force Field (CGenFF) via the CHARMM-GUI Ligand Reader & Modeler [42]. Prior to the production runs, the systems were subjected to energy minimization using the steepest descent algorithm. Subsequently, following the standard CHARMM-GUI protocol, the systems were equilibrated for a total of 1.875 ns through a stepwise relaxation process. This included sequential NVT and NPT ensembles at 300 K and 1 bar, during which position restraints on the protein and lipids were gradually released to stabilize the system [23,40]. The final production MD simulations were performed for 100 ns with a time step of 2 fs using GROMACS 2023.3. Long-range electrostatic interactions were calculated using the Particle-Mesh Ewald (PME) method, and all bonds involving hydrogen atoms were constrained using the LINCS algorithm [43]. Trajectory coordinates were saved every 20 ps for subsequent analysis.

## Results

### Virtual screening and identification of potential hits

**Fig 1:**
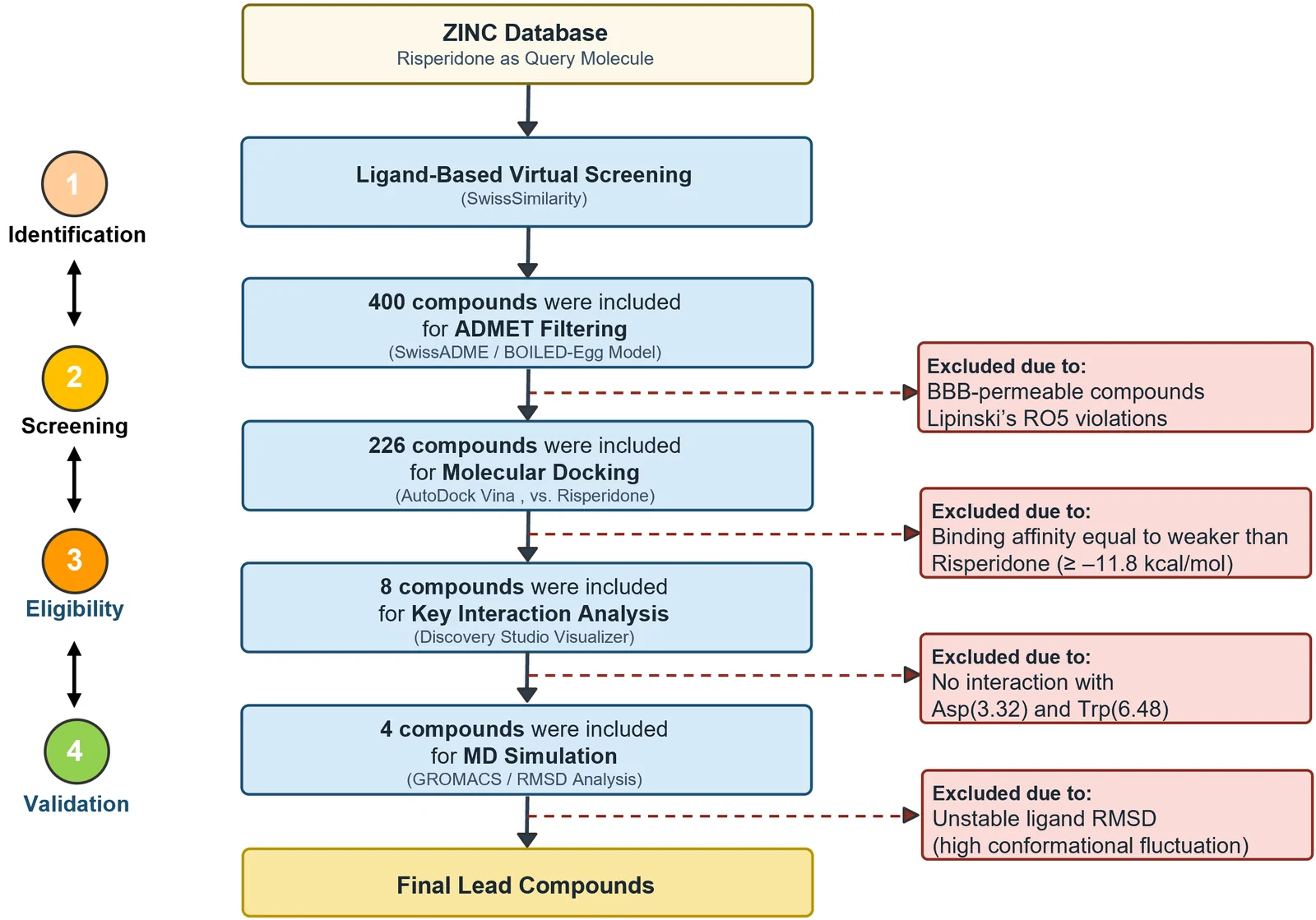
Schematic representation of the multi-stage virtual screening workflow for identifying novel peripherally restricted D2 receptor antagonists. The screening process consisted of five sequential steps: SwissSimilarity-based virtual screening of risperidone analogs, SwissADME BOILED-Egg filtering for BBB-impermeable candidates with high gastrointestinal absorption, AutoDock Vina docking against D2R, Discovery Studio-based interaction analysis focused on Asp(3.32) and Trp(6.48), and GROMACS-based MD simulation to assess conformational stability.

To identify novel D2 receptor antagonist candidates predicted to have restricted BBB penetration, a comprehensive multi-stage virtual screening workflow was employed (Fig 1). Initially, ligand-based virtual screening using the SwissSimilarity platform identified 400 structural analogs of risperidone from the ZINC-20 database. Each analog was assigned a sequential compound number (#1–#400) based on the SwissSimilarity similarity ranking, and the complete list with corresponding ZINC IDs is provided in S1 Table. These initial hits were subsequently subjected to ADMET profiling using SwissADME to filter out compounds with unfavorable pharmacokinetic properties**.** Specifically, compounds were strictly excluded if they were predicted to be BBB-permeable or if they violated Lipinski’s Rule of Five (RO5). This filtering step successfully narrowed the library down to 226 candidates exhibiting optimal gastrointestinal absorption and restricted CNS penetration.

Next, to evaluate the binding affinities of these candidates, molecular docking was performed using AutoDock Vina. This structure-based screening identified 8 top-ranking compounds that demonstrated binding energies superior to the reference drug risperidone (≤ −11.8 kcal/mol). Following the energetic evaluation, visual inspection of the binding modes confirmed that 4 out of these 8 candidates maintained critical interactions with the key orthosteric residues of the D2 receptor. Finally, to validate the dynamic stability of these protein-ligand complexes, the top 4 candidates were subjected to 100 ns molecular dynamics (MD) simulations.

### Pharmacokinetic profiles and binding affinities of top candidates

To ensure peripheral selectivity and minimize CNS-mediated side effects, the initial screening primarily focused on identifying blood-brain barrier (BBB) impermeable candidates. Based on the physicochemical descriptors of the BOILED-Egg model including WLOGP and TPSA, 226 compounds were identified as peripherally restricted agents. From this refined pool, we further narrowed the selection by prioritizing candidates that exhibited stronger binding affinity (lower binding energy) compared to risperidone.

Consequently, eight top-ranked compounds were selected based on their binding affinities to the D2 receptor. As presented in Table 2, all candidates exhibited docking scores superior to that of risperidone (−11.8 kcal/mol), with Compound #17 achieving the highest affinity (−12.4 kcal/mol). Each compound satisfied Lipinski’s Rule of Five with zero violations, and demonstrated high gastrointestinal absorption coupled with no predicted BBB permeability, supporting their potential as peripherally restricted agents. Key physicochemical descriptors, including TPSA and WLOGP, further confirmed favorable drug-likeness profiles. Synthetic accessibility scores ranged from 3.43 to 5.05, indicating that all candidates are reasonably amenable to chemical synthesis. Comprehensive ADMET profiling, detailed physicochemical data, and full SMILES strings for the entire library of 400 initial hits are provided in S1 Table.

**Table 2:** Physicochemical properties, ADMET profiles, and binding affinities of the top 8 peripherally restricted D2 receptor antagonist candidates.

Compound Number	Binding affinity (kcal/mol)	ZINC ID	Lipinski violations	TPSA	WLOGP	GI absorption	BBB permeant	Synthetic Accessibility
**Compound #17**	−12.4	ZINC000067665355	0	90.46	3.77	High	No	4.02
**Compound #21**	−11.9	ZINC000067664812	0	81.15	4.66	High	No	4.84
**Compound #24**	−11.9	ZINC000067664809	0	70.15	5.12	High	No	5.03
**Compound #27**	−11.9	ZINC000095479447	0	81.23	3.99	High	No	3.43
**Compound #128**	−12.3	ZINC000019333157	0	86.37	1.21	High	No	3.91
**Compound #321**	−12.3	ZINC000020762436	0	78.68	3.49	High	No	5.05
**Compound #360**	−11.9	ZINC000019333162	0	86.37	1.21	High	No	3.91
**Compound #381**	−11.9	ZINC000019337359	0	82.19	1.57	High	No	4.28

Binding affinities were calculated using AutoDock Vina. Risperidone was used as the reference compound with a baseline docking score of −11.8 kcal/mol. Candidates were selected based on docking scores superior to risperidone (<−11.8 kcal/mol). Compliance with Lipinski’s Rule of Five indicates zero violations, suggesting favorable oral bioavailability and drug-likeness. Synthetic accessibility scores were estimated using SwissADME, with values ranging from 1 (easy to synthesize) to 10 (difficult to synthesize). Abbreviations: ADMET, absorption, distribution, metabolism, excretion, and toxicity; BBB, blood–brain barrier; GI, gastrointestinal; TPSA, topological polar surface area; WLOGP, Wildman–Crippen octanol–water partition coefficient.

Additional computational predictions were performed to strengthen the assessment of peripheral restriction and hERG-related safety profiles of the shortlisted candidates. BBB-related parameters were evaluated for risperidone and the four shortlisted candidates using pkCSM and ADMETlab 3.0. As summarized in Table 3, risperidone showed a BBB-permeable profile, consistent with its known CNS activity. Among the shortlisted candidates, Compounds #128, #360, and #381 showed overall BBB restriction supported by additional prediction models, whereas Compound #27 showed conflicting results across platforms. In addition, potential cardiotoxicity associated with hERG potassium channel inhibition was assessed using pkCSM and ADMETlab 3.0.

**Table 3:** Additional in silico prediction of BBB restriction for risperidone and shortlisted candidates. Additional BBB-related predictions were performed using SwissADME, pkCSM, and ADMETlab 3.0.

Compound	SwissADME BBBpermeant	pkCSM BBBpermeability (logBB)	pkCSM CNSpermeability (logPS)	pkCSM P-gpsubstrate	ADMETlabBBB probability	ADMETlabPgp-substrate probability
Risperidone	Yes	0.387	−2.148	Yes	1	0.953
Compound #27	No	−1.085	−3.131	No	1	0.057
Compound #128	No	−0.666	−2.698	Yes	0.004	0.439
Compound #360	No	−0.666	−2.698	Yes	0.004	0.102
Compound #381	No	−1.055	−3.098	Yes	0.096	0.03

SwissADME BBB permeability was based on the BOILED-Egg model. pkCSM provides BBB permeability as logBB, CNS permeability as logPS, and categorical P-glycoprotein substrate prediction. ADMETlab 3.0 provides the predicted probability of BBB penetration and P-glycoprotein substrate classification, where higher values indicate a greater probability of being BBB-permeable or a P-glycoprotein substrate, respectively. Overall BBB restriction was assigned based on the combined results from SwissADME, pkCSM, and ADMETlab 3.0. Abbreviations: BBB, blood–brain barrier; CNS, central nervous system; P-gp, P-glycoprotein.

### Molecular interaction analysis and selection of final candidates

**Fig 2:**
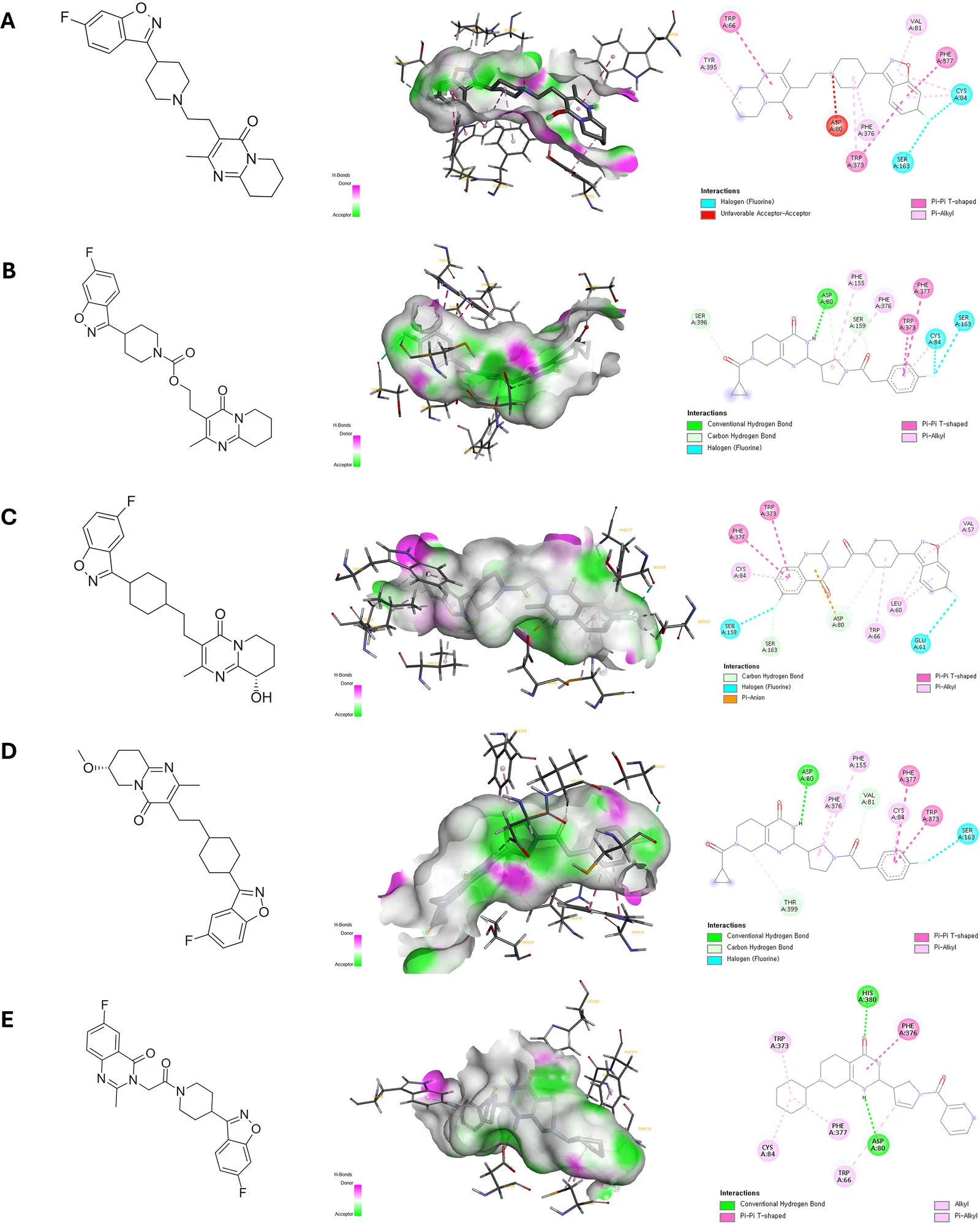
2D structures and interaction diagrams of the reference antagonist and selected hit compounds within the D2R orthosteric binding site. The 2D structures, docking poses, and interaction profiles of (A) risperidone (8NU), (B) Compound #27, (C) Compound #128, (D) Compound #360, and (E) Compound #381 are shown. Key interacting residues are labeled, and interaction types are color-coded according to the Discovery Studio scheme. All four selected candidates preserved interactions with Asp80 and Trp373, corresponding to Asp80 (3.32) and Trp373 (6.48), respectively.

To elucidate the structural basis of the binding affinities observed in the docking simulations, the binding modes of the eight top-ranked compounds were visually inspected using Discovery Studio Visualizer. The specific amino acid residues involved in the ligand-receptor interactions, along with their ZINC IDs, are summarized in Table 4, while their detailed 2D chemical structures and binding poses are illustrated in Fig 2 and S1 Fig. Prior to analyzing the new candidates, the reliability of our docking protocol was validated by examining the interaction pattern of the reference antagonist, risperidone. The docking result successfully reproduced the similar interaction pattern observed in the crystal structure (PDB: 6 CM4). While the specific types of binding interactions in our docking model showed minor deviations from the crystal structure due to differences in protonation states determined by the best docking pose, the overall binding mode remained highly consistent. Risperidone engaged a hydrophobic network that reproduced 4 of the 7 key interaction residues reported in the crystal structure, including Cys84 (3.36), Ser163 (5.46), Trp373 (6.48), and Phe377 (6.52), and maintained the critical contact with Asp80 (3.32). Asp80 (3.32) and Trp373 (6.48) are the two residues essential for D2R binding affinity [22,39], confirming the reliability of our docking protocol (Table 4 and Fig 2A).

**Table 4:** Key interacting residues of risperidone and the top hit compounds within the D2 receptor binding pocket.

Compound Number	Zinc ID	D2 Receptor Interacting Residues
**Risperidone**	**Reference**	TRP66, **Asp80**, Val81, Cys84, Ser163, **Trp373**, Phe376, Phe377, Tyr395
**17**	ZINC000067665355	Val57, Leu60, Val81, Cys84, Ser163, **Trp373**, Phe376, Phe377, Trp400
**21**	ZINC000067664812	Trp66, Val81, Cys84, **Trp373**, Phe376
**24**	ZINC000067664809	Val57, Leu60, Glu61, Trp66, Val81, Cys84, Ser163, **Trp373**, Phe376, Phe377, Thr399, Trp400
**27**	ZINC000095479447	**Asp80,** Cys84, Phe155, Ser159, Ser163, **Trp373**, Phe376, Phe377, Ser396
**128**	ZINC000019333157	Val57, Leu60, Glu61, Trp66, **Asp80**, Cys84, Ser159, Ser163, **Trp373**, Phe377
**321**	ZINC000020762436	Cys84, Val81, Ser163, **Trp373**, Trp400, Leu60, Phe376
**360**	ZINC000019333162	**Asp80**, **Trp373**, Phe376, Phe377, Phe155, Val81, Ser163, Cys84, Thr399
**381**	ZINC000019337359	**Asp80**, His380, **Trp373**, Phe376, Phe377, Trp66, Cys84

The specific interacting amino acid residues were identified based on the optimal docking poses using BIOVIA Discovery Studio Visualizer. Asp80 (3.32) and Trp373 (6.48) are highlighted in bold as the essential binding residues within the D2R orthosteric pocket.

Comparative interaction analysis identified four candidates, Compounds #27, #128, #360, and #381, that satisfied the predefined structural criteria described above. These compounds preserved the essential Asp80/Trp373-centered interaction pattern while retaining partial overlap with the conserved hydrophobic pocket residues of the risperidone-bound D2R structure (Fig 2 and Table 4). In contrast, Compounds #17, #21, #24, and #321 did not simultaneously satisfy these key interaction requirements or showed reduced overlap with the conserved hydrophobic network (S1 Fig and Table 4). Therefore, only Compounds #27, #128, #360, and #381 were advanced to MD simulation for dynamic stability assessment.

**Fig 3:**
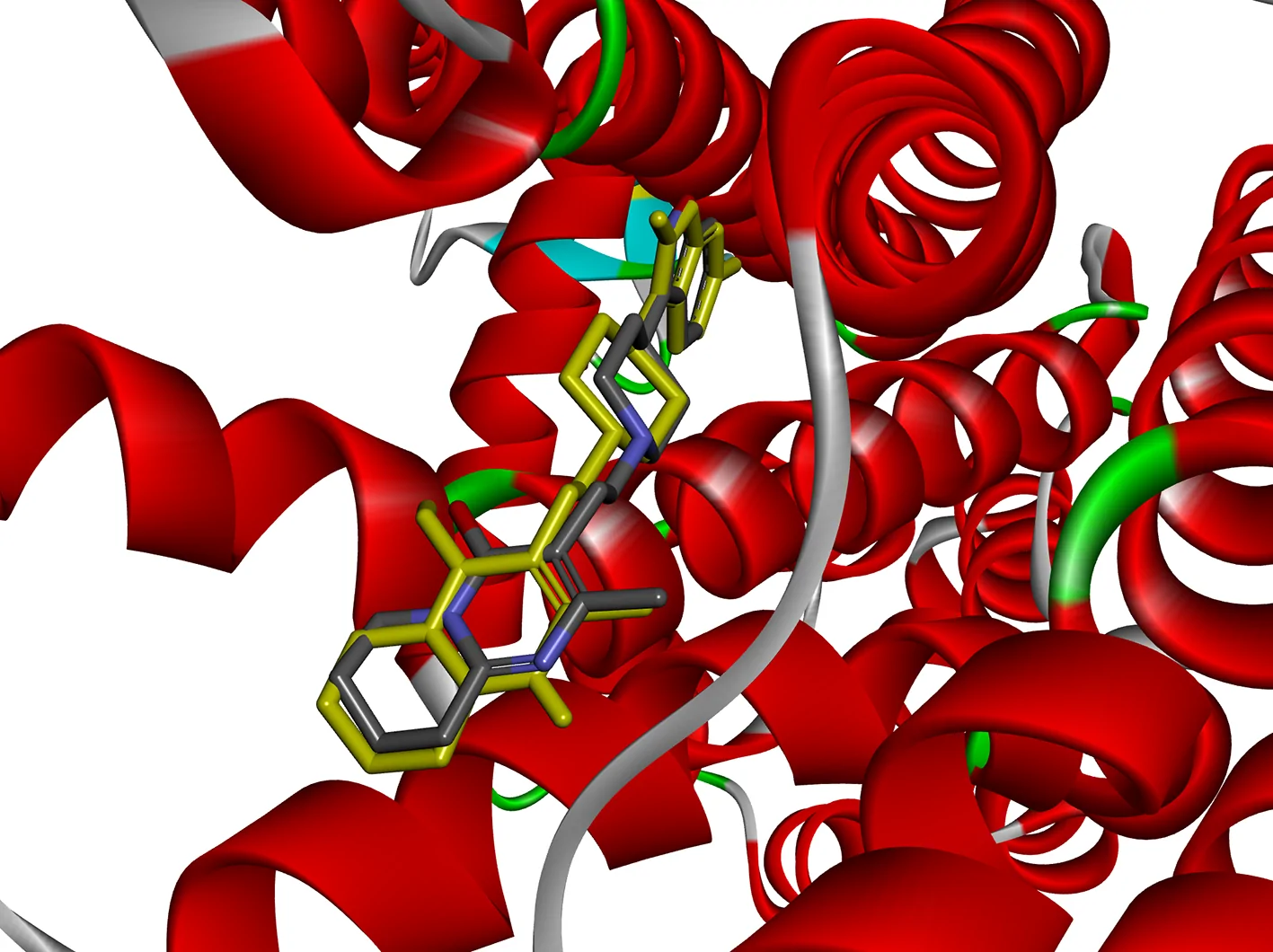
Re-docking validation of 8NU in the D2R orthosteric binding site. The redocked 8NU pose, shown in yellow, closely overlapped with the crystallographic pose, yielding an RMSD of 0.48 Å.

Molecular docking procedures were validated by re-docking the co-crystallized risperidone ligand (8NU) into the D2R orthosteric binding site (Fig 3). The re-docked pose showed good superposition with the original co-crystallized ligand, with a heavy-atom RMSD value of 0.458 Å, supporting the reliability of the docking protocol.

### MD simulation and RMSD analysis

**Fig 4:**
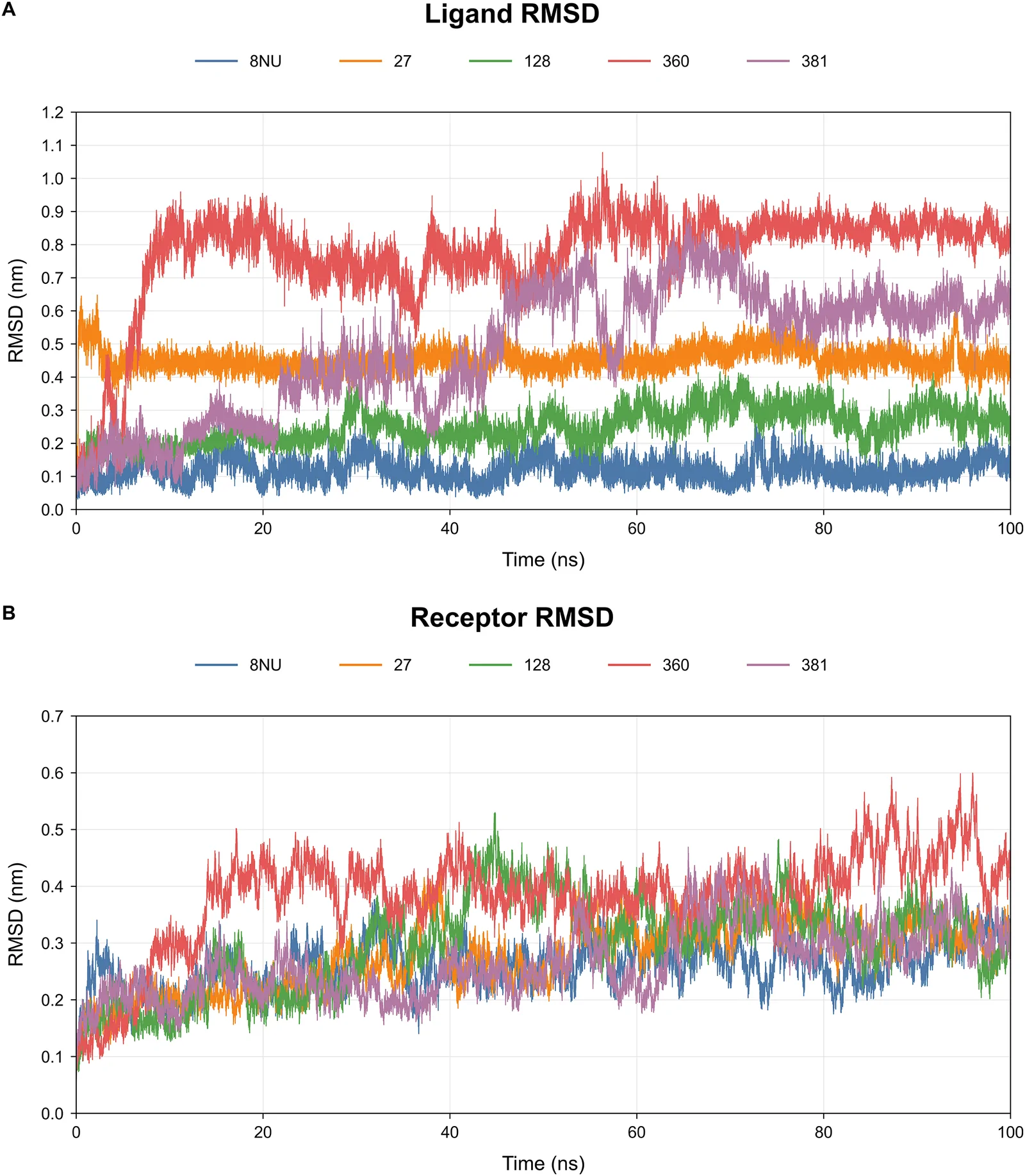
Molecular dynamics (MD) simulation analysis of D2R-ligand complexes over 100 ns. (A) Ligand RMSD profiles showing the conformational stability of the reference ligand and selected compounds within the D2R binding pocket. (B) Receptor backbone RMSD profiles showing the overall structural stability of D2R upon ligand binding. Trajectories are color-coded as follows: risperidone (8NU), blue; Compound #27, orange; Compound #128, green; Compound #360, red; and Compound #381, purple.

To validate the dynamic stability of the prioritized complexes, 100 ns molecular dynamics (MD) simulations were performed. First, the conformational stability of each compound within the binding pocket was evaluated using ligand RMSD analysis (Fig 4A). The co-crystallized reference ligand risperidone (8NU) exhibited the lowest and most stable RMSD trajectory throughout the simulation. Among the candidates, Compound #128 demonstrated the most notable ligand stability, maintaining relatively low RMSD values compared with the other candidate compounds. Compound #27 showed an intermediate RMSD profile, whereas Compound #381 displayed greater ligand mobility during the extended simulation. Conversely, Compound #360 exhibited the largest ligand displacement, suggesting less stable accommodation within the D2R binding pocket.

Subsequently, to verify the overall structural integrity and stability of the receptor upon ligand binding, the RMSD of the protein backbone (Cα atoms) was analyzed over the 100 ns MD simulation (Fig 4B). The D2 receptor complexes with the candidate compounds exhibited generally stable trajectories, with average receptor backbone RMSD values around 0.276, 0.298, 0.382, and 0.265 nm for Compounds #27, #128, #360, and #381, respectively, compared to 0.259 nm for the risperidone (8NU) complex. Although Compound #360 showed relatively higher receptor backbone fluctuation than the other systems, most of the candidate complexes maintained overall stability patterns comparable to that of the reference ligand-bound complex. This indicates that accommodation of these novel ligands does not induce severe structural instability or unfolding of the D2 receptor.

Collectively, these MD simulation results highlight Compound #128 as the most dynamically stable candidate among the four selected compounds. This compound exhibited the most favorable ligand RMSD profile among the candidates while maintaining overall receptor backbone stability comparable to the risperidone (8NU)-bound complex (Fig 4A and 4B). Based on these findings, Compound #128 was selected as the primary MD-supported lead compound for further discussion.

### Comparative ADMET and receptor docking profiles of the final candidates

To further characterize the prioritized candidates, Compounds #128 and #381 were compared with risperidone and domperidone using additional in silico profiling, including drug-likeness, absorption, hERG-related cardiotoxicity, metabolic interaction predictions, and representative aminergic receptor docking (Table 5). Both #128 and #381 satisfied major drug-likeness filters and showed high predicted gastrointestinal absorption. Compound #128 showed a synthetic accessibility score comparable to risperidone, whereas #381 was predicted to be slightly less synthetically accessible. In the absorption profile, #128 and #381 showed higher predicted human intestinal absorption and Caco-2 permeability than domperidone, while remaining comparable to or higher than risperidone.

**Table 5:** Comparative ADMET profiles of the final candidates and reference drugs.

Molecule	#128	#381	Risperidone	Domperidone
Druglikeness^(A)^				
Lipinski violations	0	0	0	0
Ghose violations	0	0	0	0
Veber violations	0	0	0	0
Egan violations	0	0	0	0
Muegge violations	0	0	0	1
Bioavailability Score	0.55	0.55	0.55	0.55
Synthetic Accessibility	3.91	4.28	3.82	2.83
Absorption^(B)^				
GI absorption	High	High	High	High
Human intestinal absorption (%)	94.28	97.66	93.84	79.07
Caco-2 permeability	1.004	1.19	0.88	0.74
hERG-related cardiotoxicity^(C)^				
hERG liability probability	0.90	0.95	0.97	0.97
hERG-10 μM probability	0.62	0.51	0.91	0.90
hERG Ⅰ inhibitor	No	No	No	Yes
hERG Ⅱ inhibitor	Yes	Yes	Yes	Yes
Metabolism^(D)^				
CYP1A2 inhibitor	0.06	0.00	0.00	0.94
	No	No	No	Yes
CYP2C19 inhibitor	0.78	0.05	0.00	0.00
	No	No	No	No
CYP2C9 inhibitor	0.99	0.00	0.00	0.00
	No	No	No	No
CYP2D6 inhibitor	0.99	1.00	1.00	1.00
	No	No	Yes	Yes
CYP3A4 inhibitor	0.63	0.99	0.00	0.00
	No	No	Yes	No
CYP2D6 substrate	0.00	0.95	1.00	0.65
	No	Yes	Yes	No
CYP3A4 substrate	0.97	0.99	1.00	1.00
	Yes	Yes	Yes	No
Off-target receptor docking (kcal/mol)^(E)^				
5-HT2A receptor	−7.9	−7.4	−7.7	−7.7
Histamine H1 receptor	−7.8	−8.7	−8.5	−8.7
α1B-adrenergic receptor	−11.2	−10	−10.4	−9.7

Compounds #128 and #381 were compared with risperidone and domperidone using SwissADME, ADMETlab 3.0, and pkCSM. (A) Drug-likeness: Lipinski, Ghose, Veber, Egan, and Muegge rule violations, bioavailability score, and synthetic accessibility were predicted using SwissADME. (B) Absorption: gastrointestinal (GI) absorption was obtained from SwissADME, whereas human intestinal absorption and Caco-2 permeability were obtained from pkCSM. (C) hERG-related cardiotoxicity: hERG liability probability and hERG-10 μM probability were obtained from ADMETlab 3.0, whereas hERG I and hERG II inhibitor classifications were obtained from pkCSM. (D) Metabolism: numerical CYP inhibitor/substrate probabilities were obtained from ADMETlab 3.0, and categorical CYP inhibitor/substrate predictions were obtained from pkCSM. Higher probability values indicate a greater predicted likelihood of the corresponding liability or interaction. (E) Off-target receptor docking: binding affinities for the 5-HT2A receptor, histamine H1 receptor, and α1B-adrenergic receptor were calculated by molecular docking.

Regarding predicted cardiotoxicity, domperidone was predicted to inhibit hERG I by pkCSM, consistent with its known hERG-related safety liability, whereas #128, #381, and risperidone were predicted to be non-inhibitors of hERG I. However, hERG II inhibition was predicted for all four compounds, and ADMETlab 3.0 indicated high overall hERG liability probabilities for #128, #381, risperidone, and domperidone. Notably, #128 showed a high hERG-10 μM probability, whereas #381 showed a lower hERG-10 μM probability than risperidone and domperidone.

In CYP-related predictions, #128 and #381 showed potential CYP interaction liabilities, although categorical predictions differed between ADMETlab 3.0 and pkCSM for several enzymes. Compound #128 showed a more favorable synthetic accessibility score than #381, whereas both candidates retained acceptable predicted absorption profiles.

Representative off-target docking against 5-HT2A, histamine H1, and α1B-adrenergic receptors showed that #128 and #381 did not uniformly increase predicted aminergic receptor binding relative to risperidone and domperidone. The candidates showed comparable 5-HT2A binding affinities (−7.4 to −7.9 kcal/mol), whereas #381 showed H1 binding comparable to domperidone and #128 showed the strongest predicted α1B-adrenergic receptor binding (−11.2 kcal/mol). These findings support #128 and #381 as prioritized candidates for further experimental testing, while emphasizing the need for *in vitro* hERG, metabolic, and off-target receptor binding assays.

## Discussion

In this study, we implemented a systematic, multi-stage in silico screening workflow to identify dopamine D2 receptor (D2R) antagonist candidates predicted to have restricted BBB penetration. By leveraging ligand-based virtual screening, ADMET-based pharmacokinetic filtering, molecular docking, and molecular dynamics (MD) simulations, we prioritized Compounds #128 and #381 as complementary candidates. Compound #128 exhibited the most favorable ligand RMSD profile among the tested candidates while maintaining overall receptor Cα stability comparable to that of the risperidone-bound complex. In contrast, Compound #381 showed greater ligand mobility but retained favorable predicted BBB restriction and a comparatively lower predicted hERG-10 μM inhibition probability than risperidone and domperidone. Both compounds maintained key docking contacts with the orthosteric residues Asp80 (3.32) and Trp373 (6.48).

A central design rationale of our approach was to exploit the unique anatomical positioning of prolactin-secreting pituitary lactotrophs, which reside outside the blood-brain barrier (BBB). While conventional antipsychotics like risperidone cross the BBB and induce central side effects, peripherally restricted agents can selectively modulate lactotroph dopaminergic signaling. The BOILED-Egg predictive model confirmed that our final candidates possess appropriate TPSA and WLOGP profiles consistent with BBB impermeability, supporting their potential as safe, peripherally acting prolactin secretagogues. In this regard, domperidone has served as a clinical precedent demonstrating that peripheral D2 antagonism is sufficient to elevate systemic prolactin levels without central dopaminergic interference. However, despite its established peripheral selectivity, the clinical utility of domperidone is severely compromised by cardiotoxicity, specifically QT prolongation and sudden cardiac death, arising from the blockade of the human ether-à-go-go-related gene (hERG) potassium channel [44–46]. These safety concerns have led the US FDA and other global regulatory agencies to impose strict restrictions on its use [47–50]. This underscores the urgent clinical need for novel peripherally restricted D2 antagonists with improved cardiac safety profiles.

Consistent with the interaction criteria defined in the docking analysis, Compounds #128 and #381 retained the key D2R binding-site contacts while showing complementary strengths across the MD and safety-oriented profiling results. Compound #128 showed the most favorable ligand RMSD profile among the tested candidates, whereas Compound #381 exhibited low receptor backbone RMSD and a comparatively lower predicted hERG-10 μM inhibition probability than risperidone and domperidone. Rather than relying solely on docking affinity, the combined interaction, MD, and ADMET results therefore support #128 and #381 as prioritized candidates for subsequent validation.

Several limitations must be acknowledged to contextualize these findings. First, the molecular docking and MD simulation approaches employed in this study primarily evaluate binding affinity and conformational stability rather than the functional outcome of ligand-receptor interactions. These methods have inherent limitations in distinguishing between agonist and antagonist binding modes, and therefore further computational approaches such as metadynamics or enhanced sampling methods, combined with functional assays, will be necessary to confirm the antagonistic activity of the identified candidates. Notably, the top candidate compounds, including Compound #128 (ZINC000019333157) and Compound #381 (ZINC000019337359), are commercially available through the ZINC database, which provides direct links to chemical vendors, thereby enabling immediate procurement for experimental validation. In vitro dopamine and prolactin signaling assays can be conducted to verify the predicted D2R binding affinity and to confirm whether these compounds functionally stimulate prolactin secretion and activate prolactin-mediated pro-survival signaling cascades. Furthermore, building upon our previous findings that risperidone attenuated DAB-induced cognitive impairment through prolactin-mediated suppression of neuroinflammatory pathways, including the TREM-1/DAP12/NLRP3/caspase-1/IL-1β and TLR4/NF-κB axes, in the mouse hippocampus [14], in vivo studies can be designed to directly compare the neuroprotective efficacy of these novel compounds against risperidone using behavioral and molecular biological analyses. Importantly, because the candidates identified in this study are peripherally restricted by design, such comparative studies would provide critical mechanistic insight into whether the cognitive protection previously observed with risperidone was mediated primarily through central D2R blockade or through peripheral lactotroph-derived prolactin signaling across the BBB.

Second, although representative off-target receptor docking and hERG-related predictions were additionally performed in this study, these analyses remain computational and require experimental confirmation. Risperidone, the parent scaffold, is known to interact with multiple monoamine receptors, including serotonin 5-HT2A, adrenergic α1, and histamine H1 receptors, and off-target binding of the identified candidates to these receptors could introduce unintended pharmacological effects. In our representative off-target docking analysis, #128 and #381 did not uniformly show stronger predicted binding to 5-HT2A, H1, and α1B-adrenergic receptors than risperidone or domperidone. However, #381 showed H1 receptor binding comparable to domperidone, and #128 showed relatively strong predicted α1B-adrenergic receptor binding, indicating that receptor selectivity should be experimentally validated. Additionally, although #381 showed a lower predicted hERG-10 μM inhibition probability than risperidone and domperidone, high overall hERG liability probabilities and predicted hERG II inhibition were observed across the tested compounds. Therefore, radioligand binding assays for monoamine receptor selectivity profiling and Human Embryonic Kidney 293-based hERG channel assays, which are standard preclinical safety assessments for second-generation antipsychotics, would be informative in addressing these concerns [51,52].

Third, the clinical application of peripherally restricted D2 antagonists for prolactin modulation requires precise and careful adjustment of prolactin levels. Our previous studies, alongside clinical observations in patients with neurodegenerative diseases, reveal that serum prolactin levels fluctuate significantly depending on the specific disease context, indicating that the relationship between prolactin and cognitive function is complex and non-linear [53]. Rather than being uniformly beneficial, the effects of prolactin on cognition may potentially be modulated by age, sex, and the underlying pathological conditions, suggesting that an optimal therapeutic window exists. Excessive prolactin elevation resulting from potent D2 antagonism can lead to hyperprolactinemia, which is clinically associated with adverse effects including reproductive dysfunction, galactorrhea, headache and visual field defects [54].

Therefore, the ideal peripherally restricted D2 antagonist should not aim to maximize prolactin secretion but rather achieve an optimal dopamine-prolactin balance within a therapeutically beneficial range. Future dose-response studies will be essential to determine the optimal dosing regimen for the identified candidates, ensuring that prolactin levels are elevated sufficiently to confer neuroprotective effects while remaining below the threshold associated with hyperprolactinemia-related complications.

## Conclusion

In this study, a systematic multi-stage in silico screening workflow integrating ligand-based virtual screening, ADMET profiling, molecular docking, molecular dynamics simulations, and safety-oriented computational profiling was employed to identify novel dopamine D2 receptor antagonist candidates predicted to have restricted BBB penetration. From 400 risperidone analogs, Compounds #128 and #381 emerged as prioritized candidates with complementary computational profiles. Compound #128 exhibited the most favorable ligand RMSD profile among the MD-tested candidates, whereas Compound #381 showed favorable predicted BBB restriction and a comparatively lower predicted hERG-10 μM inhibition probability than risperidone and domperidone. Both compounds maintained key docking contacts with residues within the D2R orthosteric binding site. Additional ADMET and representative off-target docking analyses further supported their prioritization, although potential hERG, metabolic, and receptor selectivity liabilities require experimental confirmation. These findings provide a computational rationale for advancing Compounds #128 and #381 for future evaluation as D2R antagonist candidates with predicted restricted BBB penetration. Future in vitro and in vivo studies are required to confirm D2R antagonism, prolactin secretion, BBB exclusion, receptor selectivity, cardiac safety, metabolic liability, and neuroprotective efficacy, and to determine whether peripheral prolactin elevation alone can reproduce the neuroprotective effects previously observed with centrally active D2R antagonists.

## Supporting information

S1 Fig 2D structures and interaction diagrams of the unselected hit compounds within the D2R orthosteric binding site. The 2D structures, docking poses, and interaction profiles of the excluded compounds, (A) Compound #17, (B) Compound #21, (C) Compound #24, and (D) Compound #321, are shown. These candidates were excluded because they did not simultaneously maintain interactions with Asp80 and Trp373 or showed reduced overlap with the conserved hydrophobic network. Key residues are labeled, and interaction types are color-coded as shown in the figure.
(TIF)

S2 Fig Root-mean-square fluctuation (RMSF) of Cα atoms for the D2 receptor complexes over 100 ns molecular dynamics simulations. RMSF values represent the per-residue flexibility of the receptor backbone upon ligand binding. All candidate compounds exhibited fluctuation profiles highly comparable to that of the risperidone-bound reference system, suggesting that the accommodation of these novel ligands does not significantly alter the dynamic behavior of the D2 receptor. Trajectories are color-coded as follows: risperidone (black), Compound #27 (red), Compound #128 (purple), Compound #360 (blue), and Compound #381 (green).
(TIF)

S1 Table Physicochemical properties and ADME profiles of 400 risperidone analogs identified through ligand-based virtual screening. This table presents the comprehensive physicochemical descriptors and ADMET (Absorption, Distribution, Metabolism, Excretion, and Toxicity) parameters for the 400 initial candidates identified from the ZINC-20 database using SwissSimilarity. Key parameters include molecular weight (MW), lipophilicity (Consensus Log P), topological polar surface area (TPSA), and blood-brain barrier (BBB) permeability. These data served as the primary filter to select peripherally restricted candidates for subsequent molecular docking analysis.
(XLSX)

S2 Table Complete D2R docking results for the 226 candidates retained after pharmacokinetic filtering. This table contains the AutoDock Vina docking scores, ZINC IDs, molecular structures, and selection status of the candidates subjected to D2R docking. The docking score of the crystallographic reference ligand 8NU is also provided for comparison.
(XLSX)

S1 Data Numerical source data underlying the ligand and receptor Cα RMSD plots in Fig 4. This dataset contains the time-resolved ligand RMSD and receptor Cα RMSD values for the D2R complexes containing risperidone, Compound #27, Compound #128, Compound #360, and Compound #381 over the 100 ns molecular dynamics simulations.
(XLSX)
