# Supplementary material for: Identification of novel peripherally restricted risperidone-like dopamine D2 receptor antagonists for prolactin-mediated neuroprotection through a multi-stage in silico screening
Source: PLoS One. 2026 Aug 28;21(8):e0355997. doi: 10.1371/journal.pone.0355997 (PMC13524263; doi:10.1371/journal.pone.0355997)
Supplement: Supplementary file 1 [file pone.0355997.s007.docx]

**Response to Reviewers**

**Manuscript ID: PONE-D-26-16779**

*Identification of novel peripherally restricted risperidone-like dopamine D2 receptor antagonists for prolactin-mediated neuroprotection through a multi-stage in silico screening*

Dear Editor and Reviewers,

We would like to thank the Academic Editor and the reviewers for their careful evaluation of our manuscript and for the constructive comments and suggestions. We have revised the manuscript accordingly. Our point-by-point responses are provided below.

**Responses to Reviewer #1**

**1. The manuscript appears to be well written with few punctuation errors. The authors are requested to perform a grammar check.**

**Response:**

We thank the reviewer for this comment. We carefully reviewed the entire manuscript and corrected grammatical, punctuation, spacing, and formatting errors throughout the text. We also revised several sentences to improve clarity and readability.

**Changes made in the manuscript:**
The manuscript was checked for grammar and punctuation throughout. Minor formatting errors were corrected, including reference spacing such as “intermediate[21]” to “intermediate [21]” in the Introduction and “non-linear[48]” to “non-linear [48]” in the Discussion.

**2. The authors are advised to use proper mathematical operators for multiplication symbol instead of letter x.**

**Response:**

We thank the reviewer for this comment. We corrected the multiplication symbol throughout the manuscript by replacing the letter “x” with the proper multiplication symbol “×” where applicable.

In the Molecular docking section, the grid box dimensions were revised from “30 x 30 x 30 Å” to “30 × 30 × 30 Å.”

**3. With regards to the MD simulation, the runtime of 50 ns is short and the authors are advised to redo the simulation for 100 ns to ensure the stability of the protein-ligand complex stabilization.**

**Response:**

We thank the reviewer for this valuable suggestion. We agree that a 50 ns MD simulation may be insufficient to evaluate the long-term stability of protein-ligand complexes. In response, we revised the Molecular dynamics simulations section in the Materials and methods to extend the production MD simulation time from 50 ns to 100 ns.

The co-crystallized 8NU-bound reference system provided a stable benchmark for comparison with the candidate complexes.

**Changes made in the manuscript:**
In the Materials and methods section, under “Molecular dynamics simulations,” the production MD simulation time was revised from 50 ns to 100 ns.

Original text:
“The final production MD simulations were performed for 50 ns with a time step of 2 fs using GROMACS 2023.3.”

Revised text:
“The final production MD simulations were performed for 100 ns with a time step of 2 fs using GROMACS 2023.3.”

The Results section and Fig. 4 were also updated using the 100 ns trajectories, including ligand RMSD and receptor backbone RMSD analyses.

**Responses to Reviewer #2**

**Major Comments**

**1. The study relies entirely on in silico approaches without experimental validation of D2 receptor antagonistic activity or prolactin secretion, which limits the translational significance of the findings. Inclusion of at least preliminary in vitro validation would substantially strengthen the conclusions.**

**Response:**

We thank the reviewer for this important comment. We agree that the lack of experimental validation limits the translational significance of the present study. Because this study was designed as an *in silico* screening study, preliminary in vitro validation of D2 receptor antagonistic activity and prolactin secretion could not be included in the current revision. To address this limitation, we revised the Discussion and Conclusion to avoid overstating the biological and therapeutic implications of the computational findings. We now clearly state that molecular docking and MD simulations evaluate predicted binding affinity and conformational stability, but do not confirm functional D2 receptor antagonism, prolactin secretion, or neuroprotective efficacy.

**Changes made in the manuscript:**

In the Discussion, we added the following limitation regarding the inability of docking and MD simulation to confirm functional D2 receptor antagonism

In the Discussion, we also added the following sentence to clarify the need for future biological validation of prolactin secretion and neuroprotective efficacy

**2. The manuscript does not adequately evaluate receptor selectivity against other monoamine receptors such as 5-HT2A, H1, or α1 adrenergic receptors, despite risperidone’s known promiscuity.**

**Response:**

We thank the reviewer for this important comment. We agree that receptor selectivity should be considered because risperidone is known to interact with multiple monoamine receptors in addition to D2R. In response, we revised the Introduction to explain the rationale for evaluating representative off-target monoamine receptors, including 5-HT2A, H1, and α1 adrenergic receptors. We also expanded the Molecular docking section of the Materials and methods to describe the receptor structures, co-crystallized reference ligands, and docking grid centers used for preliminary selectivity docking.

**Changes made in the manuscript:**

In the **Introduction**, we added the following paragraph to explain why receptor selectivity was considered.

In the **Materials and methods — Molecular docking** section, we added the following description of the receptor structures used for preliminary selectivity docking.

In the **Materials and methods — Molecular docking** section, we added the following docking grid information.

We also added the representative off-target docking results for 5-HT2A, H1, and α1B-adrenergic receptors to the Results section and Table 5.

**3. BBB impermeability was predicted solely using the BOILED-Egg model and SwissADME parameters. Additional computational approaches such as CNS MPO score, logBB prediction, or P-gp substrate analysis should be included to improve confidence in peripheral restriction.**

**Response:**

We thank the reviewer for this helpful suggestion. We agree that BBB impermeability should not be supported solely by the SwissADME BOILED-Egg model. In response, we expanded the BBB-related computational assessment by adding additional prediction approaches using pkCSM and ADMETlab 3.0. Specifically, pkCSM was used to evaluate BBB permeability as logBB, CNS permeability as logPS, and P-glycoprotein substrate prediction. ADMETlab 3.0 was also used to evaluate BBB penetration probability and P-glycoprotein substrate classification. These additional analyses were added to improve confidence in the predicted peripheral restriction of the shortlisted candidates.

**Changes made in the manuscript:**

In the **Materials and methods — In silico ADME profiling and BBB permeability analysis** section, we revised the BBB prediction strategy to include pkCSM and ADMETlab 3.0 in addition to the SwissADME BOILED-Egg model.

In the same section, we added the following explanation of the additional BBB-related prediction parameters.

In the **Results** section, we added the following description of the additional BBB prediction results.

**4. Molecular dynamics simulations were limited to 50 ns, which may not sufficiently capture long-term conformational stability of GPCR–ligand complexes.**

**Response:**

We agree that a 50 ns MD simulation may not be sufficient to assess the long-term conformational stability of GPCR–ligand complexes. In response, we revised the Molecular dynamics simulations section in the Materials and methods by extending the production MD simulation time from 50 ns to 100 ns.

**Changes made in the manuscript:**

In the **Materials and methods — Molecular dynamics simulations** section, we revised the production MD simulation time as follows:

The MD Results section, Fig. 4, and the supplementary Cα RMSF analysis were updated based on the extended 100 ns trajectories.

**5. The manuscript highlights Compound #381 as the lead candidate, but no comparative discussion is provided regarding its synthetic feasibility, predicted toxicity, or metabolic stability relative to existing drugs like domperidone or risperidone.**

**Response:**

In the revised analysis, the 100 ns MD simulations showed that Compound #128 exhibited the most stable ligand RMSD profile among the candidate compounds, whereas Compound #381 retained favorable BBB restriction and comparatively favorable hERG-related prediction. Therefore, we revised the manuscript to present Compounds #128 and #381 as prioritized candidates rather than emphasizing Compound #381 alone.

We also added Table 5 to compare #128 and #381 with risperidone and domperidone in terms of drug-likeness, absorption, hERG-related cardiotoxicity, CYP-related metabolic interaction predictions, and representative off-target receptor docking. Both candidates satisfied major drug-likeness criteria and showed high predicted gastrointestinal absorption. Compound #128 had a synthetic accessibility score comparable to risperidone, while #381 showed moderate synthetic feasibility. Although #381 showed a lower hERG-10 μM probability than risperidone and domperidone, both candidates retained predicted hERG and CYP-related liabilities that require experimental validation. Representative off-target docking further indicated that #128 and #381 did not uniformly show stronger binding to 5-HT2A, H1, and α1B-adrenergic receptors than the reference drugs.

**The Results, Discussion, and Conclusion were revised accordingly to clarify the complementary prioritization of #128 and #381 and to emphasize the need for future experimental validation.**

**6. The docking validation strategy is insufficiently described. Re-docking of risperidone with RMSD comparison to the crystal pose should be reported quantitatively to validate the docking protocol reliability.**

**Response:**

We performed re-docking validation using the co-crystallized risperidone ligand (8NU) extracted from the D2R crystal structure (PDB ID: 6CM4). The initially prepared risperidone structure generated from SMILES did not sufficiently reproduce the crystallographic pose; therefore, the co-crystallized 8NU ligand was used as a more appropriate reference for docking validation and MD comparison.

The re-docked 8NU pose closely overlapped with the crystal pose, yielding a heavy-atom RMSD of 0.458 Å. The docking score of the 8NU reference was −11.8 kcal/mol, slightly different from the previously used SMILES-derived risperidone value of −11.9 kcal/mol. We therefore revised the docking selection criterion to compounds with binding affinities more favorable than the 8NU reference. This change did not affect the selected candidates or downstream analyses, because all prioritized candidates showed docking scores better than −11.8 kcal/mol.

In addition, use of the co-crystallized 8NU ligand provided a more stable reference system in the 100 ns MD simulation, as reflected by its stable ligand RMSD trajectory. Thus, the revised 8NU-based reference improved both the docking validation and the dynamic stability benchmark for comparing the candidate compounds. The Methods, Results, and Fig. 3 have been revised accordingly.

**7. The discussion overstates the neuroprotective therapeutic potential despite lacking biological evidence that peripheral prolactin elevation alone can reproduce CNS protection without direct central D2R effects. The conclusions should be moderated accordingly.**

**Response:**

We thank the reviewer for this important comment. We agree that the neuroprotective therapeutic potential should not be overstated because the present study does not provide biological evidence that peripheral prolactin elevation alone can reproduce CNS protection without direct central D2R effects. In response, we revised the Discussion and Conclusion to moderate the interpretation of the computational findings and to clearly state that experimental validation is required.

**Changes made in the manuscript:**

In the **Discussion**, we clarified that the current in silico approaches cannot confirm functional D2R antagonism, prolactin secretion, or neuroprotective efficacy. The following limitation was added.

In the **Discussion**, we also added the following statement to clarify that future biological studies are required to determine whether the proposed prolactin-related mechanism can reproduce neuroprotective effects.

In the **Conclusion**, we revised the final interpretation to avoid overstating therapeutic potential and to explicitly acknowledge the lack of biological validation.

**Minor Comments**

**1. Several grammatical inconsistencies and spacing errors are present throughout the manuscript, such as “intermediate[21]” and inconsistent formatting around citations. Careful language editing is recommended.**

**Response:**

We thank the reviewer for pointing this out. We carefully reviewed the manuscript and corrected grammatical inconsistencies, spacing errors, and citation-spacing issues throughout the text.

**Changes made in the manuscript:**
Citation-spacing errors such as “intermediate[21]”, “SwissADME[29]”, and “non-linear[48]” were corrected to “intermediate [21]”, “SwissADME [29]”, and “non-linear [48]”, respectively.

**2. The numbering of residues using both model-based numbering and Ballesteros–Weinstein notation may confuse readers. A clearer explanatory table or schematic would improve readability.**

**Response:**

We thank the reviewer for this helpful comment. We agree that the simultaneous use of model-based residue numbering and Ballesteros–Weinstein notation may confuse readers without a clear explanation. In response, we added an explanatory table to clarify the correspondence among full-length human D2R numbering, model-based numbering used in this study, and Ballesteros–Weinstein notation. We also revised the residue notation throughout the manuscript to consistently present the model-based residue number followed by the Ballesteros–Weinstein notation in parentheses.

**Changes made in the manuscript:**

In the Materials and methods — Molecular docking section, we added the following explanatory sentence:

“For clarity, the correspondence among full-length D2R numbering, model-based numbering, and Ballesteros–Weinstein notation is summarized in Table 1.”

We also added the following table:

| **Functional role** | **Full-length D2R numbering** | **Model-based numbering used in this study** | **Ballesteros–Weinstein notation** |
| --- | --- | --- | --- |
| Conserved Asp in orthosteric site | Asp114 | Asp80 | 3.32 |
| Hydrophobic pocket residue | Cys118 | Cys84 | 3.36 |
| Hydrophobic pocket residue | Thr119 | Thr85 | 3.37 |
| Hydrophobic pocket residue | Ser197 | Ser163 | 5.46 |
| Hydrophobic pocket residue | Phe198 | Phe164 | 5.47 |
| Hydrophobic pocket residue | Phe390 | Phe376 | 6.44 |
| Conserved Trp / toggle-switch residue | Trp386 | Trp373 | 6.48 |
| Hydrophobic pocket residue | Phe391 | Phe377 | 6.52 |

**Table 1. Residue numbering correspondence for key D2R binding-site residues.** Residue numbering differs between the full-length human D2R sequence and the Swiss-Model-generated receptor model because the N-terminal region was truncated during model preparation. The model-based numbering is used throughout this manuscript, with Ballesteros–Weinstein notation provided in parentheses.

“Table 1. Residue numbering correspondence for key D2R binding-site residues.”

This table summarizes the correspondence between full-length D2R numbering, model-based numbering, and Ballesteros–Weinstein notation for key binding-site residues, including Asp114/Asp80/3.32 and Trp386/Trp373/6.48.

In the Materials and methods — Key interaction analysis section, we revised residue notation for consistency. For example, “Asp(3.32)” was revised to “Asp80 (3.32),” and “Trp(6.48)” was revised to “Trp373 (6.48).” Other key residues were also revised in the same format, including Cys84 (3.36), Thr85 (3.37), Ser163 (5.46), Phe164 (5.47), Phe376 (6.44), and Phe377 (6.52).

**3. The manuscript frequently repeats descriptions of Asp(3.32) and Trp(6.48) interactions across sections, which could be condensed to avoid redundancy.**

**Response:**

We thank the reviewer for this helpful suggestion. We agree that repeated descriptions of Asp(3.32) and Trp(6.48) interactions may reduce readability. In response, we condensed the Molecular interaction analysis section to avoid redundant candidate-by-candidate descriptions. The revised text now summarizes the shared Asp80/Trp373-centered interaction pattern of the selected candidates in a single comparative paragraph, while detailed residue-level information is referred to in Fig 2 and Table 4.

**Changes made in the manuscript:**

In the **Results — Molecular interaction analysis and selection of final candidates** section, the repeated descriptions of Asp80 (3.32) and Trp373 (6.48) interactions for each selected compound were condensed.

Original text:
“Comparative analysis revealed that among the eight initial hits, only four candidates, Compounds #27, #128, #360, and #381, consistently engaged both Asp80 (3.32) and Trp373 (6.48), the two pivotal residues that critically govern binding affinity. As detailed in Fig 2B and Table 2, Compound #27 exhibited a binding mode highly analogous to risperidone, reproducing 4 of the 7 key hydrophobic interactions (Cys84, Ser163, Trp373, and Phe377) while preserving the hydrogen bond with Asp80 (3.32). Compound #128 (Fig 2C) also secured the Asp80 (3.32) and Trp373 (6.48) contacts, recovering the same 4 hydrophobic residues (Cys84, Ser163, Trp373, and Phe377). Similarly, Compound #360 (Fig 2D) maintained both essential contacts with Asp80 (3.32) and Trp373 (6.48) while reproducing 4 of the 7 key hydrophobic interactions including Cys84, Ser163, Trp373, and Phe377. Compound #381 (Fig 2E) likewise preserved the Asp80 (3.32) and Trp373 (6.48) contacts, engaging 3 of the 7 key hydrophobic residues comprising Cys84, Trp373, and Phe377. In contrast, the remaining four candidates (Compounds #17, #21, #24, and #321) failed to simultaneously engage both key residues or displayed a significantly reduced overlap with the conserved hydrophobic network (S1 Fig and Table 2). Since the maintenance of these specific interactions is crucial for stable receptor binding in a dynamic environment, only the compounds satisfying these structural criteria were advanced.”

Revised text:
“Comparative interaction analysis identified four candidates, Compounds #27, #128, #360, and #381, that satisfied the predefined structural criteria described above. These compounds preserved the essential Asp80/Trp373-centered interaction pattern while retaining partial overlap with the conserved hydrophobic pocket residues of the risperidone-bound D2R structure (Fig 2 and Table 4). In contrast, Compounds #17, #21, #24, and #321 did not simultaneously satisfy these key interaction requirements or showed reduced overlap with the conserved hydrophobic network (S1 Fig and Table 4). Therefore, only Compounds #27, #128, #360, and #381 were advanced to MD simulation for dynamic stability assessment.”

In the **Fig 2 legend**, we also shortened the description to avoid repeating individual interaction details.

Revised text in Fig 2 legend:
“The 2D structures, docking poses, and interaction profiles of (A) risperidone (8NU), (B) Compound #27, (C) Compound #128, (D) Compound #360, and (E) Compound #381 are shown. Key interacting residues are labeled, and interaction types are color-coded according to the Discovery Studio scheme. All four selected candidates preserved interactions with Asp80 (3.32) and Trp373 (6.48).”

**4. Table 1 contains lengthy SMILES strings that reduce readability.**

**Response:**

We thank the reviewer for this helpful comment. We agree that the lengthy SMILES strings reduced the readability of the main table. In response, we removed the SMILES column from the main text table and provided the full SMILES strings in S1 Table. The revised main table now focuses on the key pharmacokinetic and docking-related parameters.

**Changes made in the manuscript:**

In the **Results — Pharmacokinetic profiles and binding affinities of top candidates** section, the original compound summary table was revised by removing the lengthy “SMILES CODE” column.

| **Compound Number** | **Binding affinity (kcal/mol)** | **ZINC ID** | **Lipinski violations** | **TPSA** | **WLOGP** | **GI absorption** | **BBB permeant** | **Synthetic Accessibility** |
| --- | --- | --- | --- | --- | --- | --- | --- | --- |
| **Compound #17** | -12.4 | ZINC000067665355 | 0 | 90.46 | 3.77 | High | No | 4.02 |
| **Compound #21** | -11.9 | ZINC000067664812 | 0 | 81.15 | 4.66 | High | No | 4.84 |
| **Compound #24** | -11.9 | ZINC000067664809 | 0 | 70.15 | 5.12 | High | No | 5.03 |
| **Compound #27** | -11.9 | ZINC000095479447 | 0 | 81.23 | 3.99 | High | No | 3.43 |
| **Compound #128** | -12.3 | ZINC000019333157 | 0 | 86.37 | 1.21 | High | No | 3.91 |
| **Compound #321** | -12.3 | ZINC000020762436 | 0 | 78.68 | 3.49 | High | No | 5.05 |
| **Compound #360** | -11.9 | ZINC000019333162 | 0 | 86.37 | 1.21 | High | No | 3.91 |
| **Compound #381** | -11.9 | ZINC000019337359 | 0 | 82.19 | 1.57 | High | No | 4.28 |

**Table 2. Physicochemical properties, ADMET profiles, and binding affinities of the top 8 peripherally restricted D2 receptor antagonist candidates.** Binding affinities were calculated using AutoDock Vina. Risperidone was used as the reference compound with a baseline docking score of −11.8 kcal/mol. Candidates were selected based on docking scores superior to risperidone (< −11.8 kcal/mol). Compliance with Lipinski's Rule of Five indicates zero violations, suggesting favorable oral bioavailability and drug-likeness. Synthetic accessibility scores were estimated using SwissADME, with values ranging from 1 (easy to synthesize) to 10 (difficult to synthesize). Abbreviations: ADMET, absorption, distribution, metabolism, excretion, and toxicity; BBB, blood–brain barrier; GI, gastrointestinal; TPSA, topological polar surface area; WLOGP, Wildman–Crippen octanol–water partition coefficient.

Original table:
The original Table 1 included a “SMILES CODE” column containing long SMILES strings for each compound.

Revised table:
The revised compound summary table no longer includes the SMILES column and now presents the key parameters, including compound number, binding affinity, ZINC ID, Lipinski violations, TPSA, WLOGP, gastrointestinal absorption, BBB permeability, and synthetic accessibility.

We also added the following statement to indicate where the full SMILES information is provided:

“Comprehensive ADMET profiling, detailed physicochemical data, and full SMILES strings for the entire library of 400 initial hits are provided in S1 Table.”

Because a new residue-numbering table was added earlier in the manuscript, the compound summary table was renumbered from Table 1 to Table 2.

**5. Figure legends are highly detailed and somewhat repetitive. Simplifying the legends while keeping essential methodological information would improve clarity.**

**Response:**

We thank the reviewer for this helpful suggestion. We agree that some figure legends were overly detailed and repetitive. In response, we simplified the legends for Fig 1, Fig 2, and S1 Fig while retaining the essential methodological information required to interpret each figure.

**Changes made in the manuscript:**

In the **Fig 1 legend**, we simplified the description of the screening process by summarizing the major steps rather than providing excessive procedural detail.

Revised text in Fig 1 legend:
“Fig 1. Multi-stage in silico screening process for identifying peripherally restricted D2R antagonist candidates. The screening process consisted of five sequential steps: SwissSimilarity-based virtual screening of risperidone analogs, SwissADME BOILED-Egg filtering for BBB-impermeable candidates with high gastrointestinal absorption, AutoDock Vina docking against D2R, Discovery Studio-based interaction analysis focused on Asp80 (3.32) and Trp373 (6.48), and GROMACS-based MD simulation to assess conformational stability.”

In the **Fig 2 legend**, we reduced repeated candidate-by-candidate interaction descriptions and retained only the essential information regarding docking poses, interaction profiles, key residues, and color-coded interaction annotations.

Revised text in Fig 2 legend:
“Fig 2. Molecular docking poses and interaction profiles of risperidone and selected candidate compounds within the D2R orthosteric binding site. The 2D structures, docking poses, and interaction profiles of (A) risperidone (8NU), (B) Compound #27, (C) Compound #128, (D) Compound #360, and (E) Compound #381 are shown. Key interacting residues are labeled, and interaction types are color-coded according to the Discovery Studio scheme. All four selected candidates preserved interactions with Asp80 (3.32) and Trp373 (6.48).”

In the **S1 Fig legend**, we shortened the explanation for the excluded compounds while retaining the essential reason for exclusion.

Revised text in S1 Fig legend:
“S1 Fig. 2D structures and interaction diagrams of the unselected hit compounds within the D2R orthosteric binding site. The 2D chemical structures, docking poses, and molecular interactions of the candidates that were excluded after interaction analysis are shown: (A) Compound #17, (B) Compound #21, (C) Compound #24, and (D) Compound #321. These candidates failed to simultaneously engage both Asp80 (3.32) and Trp373 (6.48), or displayed reduced overlap with the conserved hydrophobic network, and were therefore excluded from further analysis. Key residues are labeled, and interaction types are color-coded according to the Discovery Studio scheme.”

**6. The authors mention cardiotoxicity concerns related to hERG inhibition but do not provide any in silico hERG liability prediction for the shortlisted compounds. Including this information would strengthen the safety assessment.**

**Response:**

We thank the reviewer for this important suggestion. We agree that hERG liability prediction would strengthen the preliminary safety assessment of the shortlisted compounds. In response, we added an in silico hERG-related safety assessment for the final prioritized candidates (#128 and #381) and reference drugs using pkCSM and ADMETlab 3.0.

**Changes made in the manuscript:**

In the **Results** section, we added the following sentence describing the hERG-related prediction results:

In the **Discussion**, we clarified that the hERG prediction results do not establish cardiac safety and require further validation:

**7. The Data Availability statement appears incomplete because docking files, MD trajectories, and processed datasets are not explicitly deposited in a public repository.**

**Response:**

We revised the Data Availability statement to more explicitly describe the availability of the datasets generated and analyzed in this study. The processed virtual screening results, docking scores, ADMET prediction data, key interaction tables, and MD-derived analysis files used to generate the figures and tables are provided in the Supporting Information. Because the full MD trajectory files are large, the processed trajectory-derived data required to reproduce the reported RMSD analyses are provided with the revised submission. The Data Availability statement has been updated accordingly.

We again thank the Academic Editor and the reviewers for their valuable comments, which helped us improve the quality and clarity of the manuscript.

Sincerely,

Min-Sun Kim, Ph.D.
Corresponding Author
College of Pharmacy, Sunchon National University
