# Supplementary material for: Identification of novel peripherally restricted risperidone-like dopamine D2 receptor antagonists for prolactin-mediated neuroprotection through a multi-stage in silico screening
Source: PLoS One. 2026 Aug 28;21(8):e0355997. doi: 10.1371/journal.pone.0355997 (PMC13524263; doi:10.1371/journal.pone.0355997)
Supplement: Peer review history

# Peer review: Decision Letter 0

**DOI:** 10.1371/journal.pone.0355997.r001

**Authors:** Lalit Samant

7 May 2026

Dear Dr. Kim,

Please submit your revised manuscript by Jun 21 2026 11:59PM. If you will need more time than this to complete your revisions, please reply to this message or contact the journal office at plosone@plos.org. When you're ready to submit your revision, log on to https://www.editorialmanager.com/pone/ and select the 'Submissions Needing Revision' folder to locate your manuscript file.

- A letter that responds to each point raised by the academic editor and reviewer(s). You should upload this letter as a separate file labeled 'Response to Reviewers'.
- A marked-up copy of your manuscript that highlights changes made to the original version. You should upload this as a separate file labeled 'Revised Manuscript with Track Changes'.
- An unmarked version of your revised paper without tracked changes. You should upload this as a separate file labeled 'Manuscript'.

If you would like to make changes to your financial disclosure, please include your updated statement in your cover letter. Guidelines for resubmitting your figure files are available below the reviewer comments at the end of this letter.

If applicable, we recommend that you deposit your laboratory protocols in protocols.io to enhance the reproducibility of your results. Protocols.io assigns your protocol its own identifier (DOI) so that it can be cited independently in the future. For instructions see: https://journals.plos.org/plosone/s/submission-guidelines#loc-laboratory-protocols. Additionally, PLOS ONE offers an option for publishing peer-reviewed Lab Protocol articles, which describe protocols hosted on protocols.io. Read more information on sharing protocols at https://plos.org/protocols?utm_medium=editorial-email&utm_source=authorletters&utm_campaign=protocols.

As the corresponding author, your ORCID iD is verified in the submission system and will appear in the published article. PLOS supports the use of ORCID, and we encourage all coauthors to register for an ORCID iD and use it as well. Please encourage your coauthors to verify their ORCID iD within the submission system before final acceptance, as unverified ORCID iDs will not appear in the published article. *Only*  the individual author can complete the verification step; PLOS staff *cannot*  verify ORCID iDs on behalf of authors.

We look forward to receiving your revised manuscript.

Kind regards,

Lalit Samant

Academic Editor

PLOS One

Journal Requirements:

When submitting your revision, we need you to address these additional requirements.

1. Please ensure that your manuscript meets PLOS ONE's style requirements, including those for file naming. The PLOS ONE style templates can be found at

https://journals.plos.org/plosone/s/file?id=wjVg/PLOSOne_formatting_sample_main_body.pdf and https://journals.plos.org/plosone/s/file?id=ba62/PLOSOne_formatting_sample_title_authors_affiliations.pdf

2. Please note that PLOS One has specific guidelines on code sharing for submissions in which author-generated code underpins the findings in the manuscript. In these cases, we expect all author-generated code to be made available without restrictions upon publication of the work. Please review our guidelines at https://journals.plos.org/plosone/s/materials-and-software-sharing#loc-sharing-code and ensure that your code is shared in a way that follows best practice and facilitates reproducibility and reuse.

3. Thank you for stating the following financial disclosure:

This study supported by Sunchon National University.

Please state what role the funders took in the study. If the funders had no role, please state: "The funders had no role in study design, data collection and analysis, decision to publish, or preparation of the manuscript."

If this statement is not correct you must amend it as needed.

Please include this amended Role of Funder statement in your cover letter; we will change the online submission form on your behalf.

4. Thank you for stating the following in the Acknowledgments Section of your manuscript:

This study supported by Sunchon National University.

We note that you have provided funding information that is not currently declared in your Funding Statement. However, funding information should not appear in the Acknowledgments section or other areas of your manuscript. We will only publish funding information present in the Funding Statement section of the online submission form.

Please remove any funding-related text from the manuscript and let us know how you would like to update your Funding Statement. Currently, your Funding Statement reads as follows:

This study supported by Sunchon National University.

Please include your amended statements within your cover letter; we will change the online submission form on your behalf.

5. If the reviewer comments include a recommendation to cite specific previously published works, please review and evaluate these publications to determine whether they are relevant and should be cited. There is no requirement to cite these works unless the editor has indicated otherwise.

[Note: HTML markup is below. Please do not edit.]

Reviewers' comments:

Reviewer's Responses to Questions

**Comments to the Author**

1. Is the manuscript technically sound, and do the data support the conclusions?

Reviewer #1: Yes

Reviewer #2: No

**********

2. Has the statistical analysis been performed appropriately and rigorously?

Reviewer #1: Yes

Reviewer #2: No

**********

3. Have the authors made all data underlying the findings in their manuscript fully available?

The PLOS Data policy

Reviewer #1: Yes

Reviewer #2: Yes

**********

4. Is the manuscript presented in an intelligible fashion and written in standard English?

Reviewer #1: Yes

Reviewer #2: Yes

**********

Reviewer #1: The manuscript appears to be well written with few punctuation errors. The authors are requested to perform a grammar check. The authors are advised to use proper mathematical operators for multiplication symbol instead of letter x. With regards to the MD simulation, the runtime of 50ns is short and the authors are advised to redo the simulation for 100 ns to ensure the sability of the protein-ligand complex stabilization

Reviewer #2: Major Comments

1. The study relies entirely on in silico approaches without experimental validation of D2 receptor antagonistic activity or prolactin secretion, which limits the translational significance of the findings. Inclusion of at least preliminary in vitro validation would substantially strengthen the conclusions.

2. The manuscript does not adequately evaluate receptor selectivity against other monoamine receptors such as 5-HT2A, H1, or α1 adrenergic receptors, despite risperidone’s known promiscuity.

3. BBB impermeability was predicted solely using the BOILED-Egg model and SwissADME parameters. Additional computational approaches such as CNS MPO score, logBB prediction, or P-gp substrate analysis should be included to improve confidence in peripheral restriction.

4. Molecular dynamics simulations were limited to 50 ns, which may not sufficiently capture long-term conformational stability of GPCR–ligand complexes.

5. The manuscript highlights Compound #381 as the lead candidate, but no comparative discussion is provided regarding its synthetic feasibility, predicted toxicity, or metabolic stability relative to existing drugs like domperidone or risperidone.

6. The docking validation strategy is insufficiently described. Re-docking of risperidone with RMSD comparison to the crystal pose should be reported quantitatively to validate the docking protocol reliability.

7. The discussion overstates the neuroprotective therapeutic potential despite lacking biological evidence that peripheral prolactin elevation alone can reproduce CNS protection without direct central D2R effects. The conclusions should be moderated accordingly.

Minor Comments

1. Several grammatical inconsistencies and spacing errors are present throughout the manuscript, such as “intermediate[21]” and inconsistent formatting around citations. Careful language editing is recommended.

2. The numbering of residues using both model-based numbering and Ballesteros–Weinstein notation may confuse readers. A clearer explanatory table or schematic would improve readability.

3. The manuscript frequently repeats descriptions of Asp(3.32) and Trp(6.48) interactions across sections, which could be condensed to avoid redundancy.

4. Table 1 contains lengthy SMILES strings that reduce readability.

5. Figure legends are highly detailed and somewhat repetitive. Simplifying the legends while keeping essential methodological information would improve clarity.

6. The authors mention cardiotoxicity concerns related to hERG inhibition but do not provide any in silico hERG liability prediction for the shortlisted compounds. Including this information would strengthen the safety assessment.

7. The Data Availability statement appears incomplete because docking files, MD trajectories, and processed datasets are not explicitly deposited in a public repository.

**********

what does this mean?). If published, this will include your full peer review and any attached files.

If you choose “no”, your identity will remain anonymous but your review may still be made public.

**Do you want your identity to be public for this peer review?**  For information about this choice, including consent withdrawal, please see our Privacy Policy

Reviewer #1: No

Reviewer #2: No

**********

[NOTE: If reviewer comments were submitted as an attachment file, they will be attached to this email and accessible via the submission site. Please log into your account, locate the manuscript record, and check for the action link "View Attachments". If this link does not appear, there are no attachment files.]

To ensure your figures meet our technical requirements, please review our figure guidelines: https://journals.plos.org/plosone/s/figures

You may also use PLOS’s free figure tool, NAAS, to help you prepare publication quality figures: https://journals.plos.org/plosone/s/figures#loc-tools-for-figure-preparation.

NAAS will assess whether your figures meet our technical requirements by comparing each figure against our figure specifications.

# Author comment: Author response to Decision Letter 1

**DOI:** 10.1371/journal.pone.0355997.r002

27 Jul 2026

Dear Editor and Reviewers,

We sincerely thank the Academic Editor and reviewers for their careful evaluation of our manuscript. We have revised the manuscript in response to all comments, including extension of the MD simulations to 100 ns, re-docking validation using co-crystallized 8NU, additional BBB-related predictions, hERG and off-target receptor docking analyses, revision of the lead-candidate interpretation to prioritize Compounds #128 and #381, and moderation of the conclusions to reflect the computational nature of the study.

A detailed point-by-point response to each reviewer comment has been uploaded as a separate file labeled “Response to Reviewers.”

Sincerely,

Min-Sun Kim, Ph.D.

Corresponding Author

Attachment Submitted filename: Response to Reviewers.docx

# Editor report: Decision Letter 1

**DOI:** 10.1371/journal.pone.0355997.r003

**Authors:** Lalit Samant

28 Jul 2026

Identification of novel peripherally restricted risperidone-like dopamine D2 receptor antagonists for prolactin-mediated neuroprotection through a multi-stage in silico screening

PONE-D-26-16779R1

Dear Dr. Kim,

We’re pleased to inform you that your manuscript has been judged scientifically suitable for publication and will be formally accepted for publication once it meets all outstanding technical requirements.

Within one week, you’ll receive an e-mail detailing the required amendments. When these have been addressed, you’ll receive a formal acceptance letter and your manuscript will be scheduled for publication.

An invoice will be generated when your article is formally accepted. Please note, if your institution has a publishing partnership with PLOS and your article meets the relevant criteria, all or part of your publication costs will be covered. Please make sure your user information is up-to-date by logging into Editorial Manager at Editorial Manager® and clicking the ‘Update My Information' link at the top of the page. For questions related to billing, please contact billing support.

If your institution or institutions have a press office, please notify them about your upcoming paper to help maximize its impact. If they’ll be preparing press materials, please inform our press team as soon as possible -- no later than 48 hours after receiving the formal acceptance. Your manuscript will remain under strict press embargo until 2 pm Eastern Time on the date of publication. For more information, please contact onepress@plos.org.

Kind regards,

Lalit Samant

Academic Editor

PLOS One

Additional Editor Comments (optional):

Reviewers' comments:

# Editor report: Acceptance letter

**DOI:** 10.1371/journal.pone.0355997.r004

**Authors:** Lalit Samant

PONE-D-26-16779R1

PLOS One

Dear Dr. Kim,

I'm pleased to inform you that your manuscript has been deemed suitable for publication in PLOS One. Congratulations! Your manuscript is now being handed over to our production team.

At this stage, our production department will prepare your paper for publication. This includes ensuring the following:

* All references, tables, and figures are properly cited

* All relevant supporting information is included in the manuscript submission,

* There are no issues that prevent the paper from being properly typeset

You will receive further instructions from the production team, including instructions on how to review your proof when it is ready. Please keep in mind that we are working through a large volume of accepted articles, so please give us a few days to review your paper and let you know the next and final steps.

Lastly, if your institution or institutions have a press office, please let them know about your upcoming paper now to help maximize its impact. If they'll be preparing press materials, please inform our press team within the next 48 hours. Your manuscript will remain under strict press embargo until 2 pm Eastern Time on the date of publication. For more information, please contact onepress@plos.org.

You will receive an invoice from PLOS for your publication fee after your manuscript has reached the completed accept phase. If you receive an email requesting payment before acceptance or for any other service, this may be a phishing scheme. Learn how to identify phishing emails and protect your accounts at https://explore.plos.org/phishing.

If we can help with anything else, please email us at customercare@plos.org.

Thank you for submitting your work to PLOS ONE and supporting open access.

Kind regards,

PLOS ONE Editorial Office Staff

on behalf of

Dr. Lalit Samant

Academic Editor

PLOS One
